# Improving Dietary Supplement Information Retrieval: Development of a Retrieval-Augmented Generation System With Large Language Models

**DOI:** 10.2196/67677

**Published:** 2025-03-19

**Authors:** Yu Hou, Jeffrey R Bishop, Hongfang Liu, Rui Zhang

**Affiliations:** 1 Division of Computational Health Sciences University of Minnesota Minneapolis, MN United States; 2 Department of Experimental and Clinical Pharmacology University of Minnesota Minneapolis, MN United States; 3 Department of Health Data Science and Artificial Intelligence UTHealth Houston, TX United States; 4 Center for Learning Health System Sciences University of Minnesota Minneapolis, MN United States

**Keywords:** dietary supplements, knowledge representation, knowledge graph, retrieval-augmented generation, large language model, user interface

## Abstract

**Background:**

Dietary supplements (DSs) are widely used to improve health and nutrition, but challenges related to misinformation, safety, and efficacy persist due to less stringent regulations compared with pharmaceuticals. Accurate and reliable DS information is critical for both consumers and health care providers to make informed decisions.

**Objective:**

This study aimed to enhance DS-related question answering by integrating an advanced retrieval-augmented generation (RAG) system with the integrated Dietary Supplement Knowledgebase 2.0 (iDISK2.0), a dietary supplement knowledge base, to improve accuracy and reliability.

**Methods:**

We developed iDISK2.0 by integrating updated data from authoritative sources, including the Natural Medicines Comprehensive Database, the Memorial Sloan Kettering Cancer Center database, Dietary Supplement Label Database, and Licensed Natural Health Products Database, and applied advanced data cleaning and standardization techniques to reduce noise. The RAG system combined the retrieval power of a biomedical knowledge graph with the generative capabilities of large language models (LLMs) to address limitations of stand-alone LLMs, such as hallucination. The system retrieves contextually relevant subgraphs from iDISK2.0 based on user queries, enabling accurate and evidence-based responses through a user-friendly interface. We evaluated the system using true-or-false and multiple-choice questions derived from the Memorial Sloan Kettering Cancer Center database and compared its performance with stand-alone LLMs.

**Results:**

iDISK2.0 integrates 174,317 entities across 7 categories, including 8091 dietary supplement ingredients; 163,806 dietary supplement products; 786 diseases; and 625 drugs, along with 6 types of relationships. The RAG system achieved an accuracy of 99% (990/1000) for true-or-false questions on DS effectiveness and 95% (948/100) for multiple-choice questions on DS-drug interactions, substantially outperforming stand-alone LLMs like GPT-4o (OpenAI), which scored 62% (618/1000) and 52% (517/1000) on these respective tasks. The user interface enabled efficient interaction, supporting free-form text input and providing accurate responses. Integration strategies minimized data noise, ensuring access to up-to-date, DS-related information.

**Conclusions:**

By integrating a robust knowledge graph with RAG and LLM technologies, iDISK2.0 addresses the critical limitations of stand-alone LLMs in DS information retrieval. This study highlights the importance of combining structured data with advanced artificial intelligence methods to improve accuracy and reduce misinformation in health care applications. Future work includes extending the framework to broader biomedical domains and improving evaluation with real-world, open-ended queries.

## Introduction

Dietary supplements (DSs) are products intended to add nutritional value to the diet, often containing vitamins, minerals, herbs, amino acids, or other substances [1,2]. In recent years, the role of DSs has become increasingly recognized, with a significant portion of the population acknowledging their importance. According to surveys, 9 out of 10 DS users believe in the necessity of these products, reflecting their widespread acceptance and use [3]. DSs may aid in preventing chronic conditions and offer the potential for reducing health care costs by minimizing the occurrence or severity of preventable diseases [4]. Unlike prescription and over-the-counter drugs, which are subject to stringent regulations, DSs are classified as food products and are regulated by the Food and Drug Administration under more lenient guidelines. Many individuals use DSs spontaneously, without clinician oversight, which introduces unique challenges related to efficacy, safety, and regulation [5]. A significant concern arises from the fact that around 23,000 doctor visits per year are linked to DS-related adverse effects [6]. This underscores the need for both consumers and health care providers to have access to accurate and comprehensive resources for DS-related information.

In response to the increasing demand for reliable DS information, biomedical knowledge graphs (BKGs) have been developed over the past decade to manage large-scale, heterogeneous biomedical data [7-9]. In 2020, the integrated Dietary Supplement Knowledgebase (iDISK) was established to meet the growing need for standardized DS information [10]. iDISK aggregates data from 4 major resources: the Natural Medicines Comprehensive Database (NMCD) [11], the “About Herbs” page on the Memorial Sloan Kettering Cancer Center (MSKCC) website [12], the Dietary Supplement Label Database (DSLD) [13], and the Licensed Natural Health Products Database (LNHPD) [14]. iDISK includes over 4200 ingredient concepts connected to drugs, diseases, symptoms, therapeutic classes, organ systems, and products, providing an extensive repository of DS knowledge.

However, as DS information continually evolves—such as updates to product labels and the discovery of new ingredient-drug interactions—some aspects of the original iDISK have become outdated. For instance, since the launch of DSLD in 2013, the number of recorded labels has increased from 16,712 to 183,012 by January 2024. To maintain accuracy, it is crucial to update iDISK using the latest database versions and optimize the integration process to minimize data noise. Large language models (LLMs) have also gained popularity for answering DS-related inquiries, yet these models are prone to generating “hallucinations” or inaccurate information [15-18]. To counter this, retrieval-augmented generation (RAG) combines the retrieval power of knowledge bases with the generative capabilities of LLMs to produce more accurate responses [19]. An RAG system operates in two phases: first, it retrieves relevant data from a structured knowledge base, ensuring accuracy, and then it uses an LLM to generate responses [20,21]. By integrating iDISK with an RAG system, we mitigate the hallucination problem inherent in standalone LLMs [22,23].

In this paper, we (1) present a major update from iDISK to iDISK2.0, incorporating the latest database versions while minimizing data noise; (2) integrate an RAG system with LLMs to significantly improve the accuracy of DS question answering, the first of its kind for DS queries; and (3) develop the integrated Dietary Supplement Knowledgebase—retrieval-augmented generation (iDISK2.0-RAG) web portal, which offers consumers and health care providers a reliable and user-friendly interface for DS information.

Our work not only addresses the limitations of LLMs but also establishes a new standard for DS question answering. Although this work focuses on DSs, our successful integration and platform can provide new opportunities for other domains.

## Methods

### Overview

**Figure 1:**
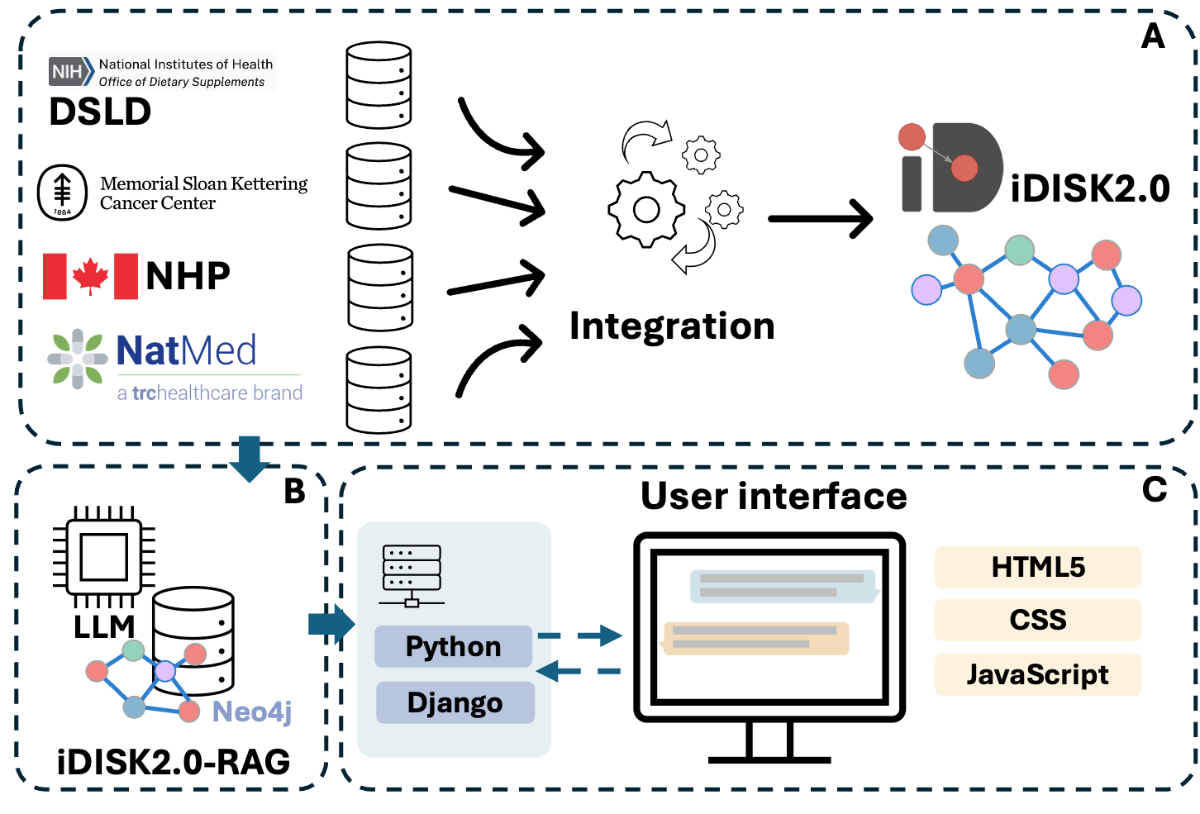
An illustration of the study pipeline. DSLD: Dietary Supplement Label Database; NHP: Natural Health Products; iDISK2.0: integrated Dietary Supplement Knowledgebase; LLM: large language model; iDISK2.0-RAG: integrated Dietary Supplement Knowledgebase—retrieval-augmented generation.

Figure 1 illustrates the overall workflow of this study. We first constructed iDISK2.0 by integrating the latest versions of data from 4 DS resource databases that were originally integrated into iDISK, including LNHPD, DSLD, MSKCC, and NMCD (Figure 1A). We further developed an RAG-based LLM on iDISK2.0 (Figure 1B). In addition, we developed a user-friendly intelligent user interface with iDISK2.0-RAG as the backend to answer DS-related questions (Figure 1C).

### Data Collection and Preprocessing

A total of 4 datasets were collected and preprocessed for the iDISK 2.0. Data from DSLD were downloaded directly from the data release, and the DSLD application programming interface (API) was used to enrich the dietary supplement-related information. Data from MSKCC were obtained after approval, using a web crawler to extract relevant information. LNHPD data were downloaded directly from the data release. After acquiring information from all sources, we converted them into a unified, structured format. The data of the NMCD used in the iDISK 2.0 were derived from information that already exists within iDISK. During the information extraction process, we used LLM (GPT-4) with the prompt (see Multimedia Appendix 1 for specific prompts) to assist in extracting and optimizing information. For example, relationships between DSs and diseases recorded in MSKCC were expressed in text format; we used the LLM to extract disease and drug entities from these sentences and organize them into a structured format for integration and processing. The source data contained inconsistencies and noise, such as variations in how company addresses were recorded in the DSLD product data (eg, “U.S.A.,” “United States of America,” and “United States”) and nonsensical ingredient names like “8” or “%.” We addressed these issues by using an LLM to identify and unify the differing expressions. In addition, we developed filtering patterns specifically designed to remove nonsensical ingredient names, such as those with fewer than two characters or those consisting solely of numbers or punctuation. This approach ensured a more consistent and accurate dataset by standardizing key information and eliminating irrelevant entries.

Specifically, we extracted the dietary supplement product (DSP) name, purpose, safety information, and company address information from LNHPD, along with the dietary supplement ingredient (DSI) name, source material information, and relationships between DSP and DSI. From DSLD, we extracted the DSP name, company address information, DSI name, and the relationship between DSP and DSI. In DSLD, names of DSIs appeared as duplicate records due to synonyms (eg, “Fiber gum acacia,” “Acacia gum extract,” and “Acacia”). We used the DSLD API to collate these synonyms and eliminated duplicates by grouping the synonyms of DSIs representing the same entity using the “Ingredient Group” classification in DSLD. From MSKCC, we extracted the DSI name, common name, background information, mechanism of action, disease name, drug name, symptom name, and the relationships between DSI and disease, DSI and symptom, and DSI and drug.

After completing the information extraction and optimization, we used QuickUMLS (Georgetown IR Lab) [24] to map DSI entities, symptom entities, disease entities, and drug entities from the source resources to the Unified Medical Language System (UMLS) [25] Concept Unique Identifier (CUI). To ensure mapping accuracy, we restricted each entity type based on UMLS semantic types (see Multimedia Appendix 1). The results of the QuickUMLS mapping included the matched UMLS CUI, term name, similarity, and whether the term was preferred. When QuickUMLS returned multiple results, we used a prioritization strategy: first, results with a similarity of 1, marked as preferred; next, results with a similarity of 1, even if not marked as preferred; and finally, those marked as preferred with the highest similarity. If none of these conditions were met, a UMLS CUI was not assigned to the entity to ensure mapping accuracy.

### Data Integration for IDISK2.0

We used a greedy strategy to standardize entity terminology and integrate data from different sources. For each specific entity type, we initially selected a single database to initialize the entity vocabulary. Each specific entity was linked with a unique identifier to integrate entities from all databases, progressively refining the entity vocabulary. Specifically, for DSP, we started with data from LNHPD, integrating DSP entities from both LNHPD and DSLD. We consolidated DSP with the same product name and company name into a single unique entity. We unified entities with the same UMLS CUI or identical term names for DSI. Similarly, we consolidated entities with the same UMLS CUI or identical term names for disease, symptom, and drug entities. Each integrated entity was assigned a unique iDISK ID as its identifier in iDISK. Following this normalization procedure, we obtained a CSV file for each entity type, storing all standardized entity terms and their associated attributes. This allowed us to integrate the extracted knowledge from different databases to build iDISK2.0.

In the knowledge graph (KG), the basic knowledge unit is a triplet, typically defined as “<head entity, relation, tail entity>,” indicating a relationship from the head entity to the tail entity in the KG. We mapped the head and tail nodes of the knowledge from the source databases to iDISK IDs, establishing triples composed of iDISK IDs and their relations. We then deduplicated all these triples to reduce noise in iDISK while ensuring quality. Finally, multiple rounds of manual quality checks were conducted.

### iDISK2.0 Deployment with Neo4j

We deployed the iDISK2.0 using Neo4j [26], a well-designed graph database platform that allows for structured queries within graphs. Specifically, we stored the generated entities and relationships of iDISK in corresponding CSV files, which Neo4j used as input to create a KG instance automatically. This setup enables iDISK2.0 to be efficiently and flexibly interacted with and updated.

### iDISK2.0-Based RAG System

**Figure 2:**
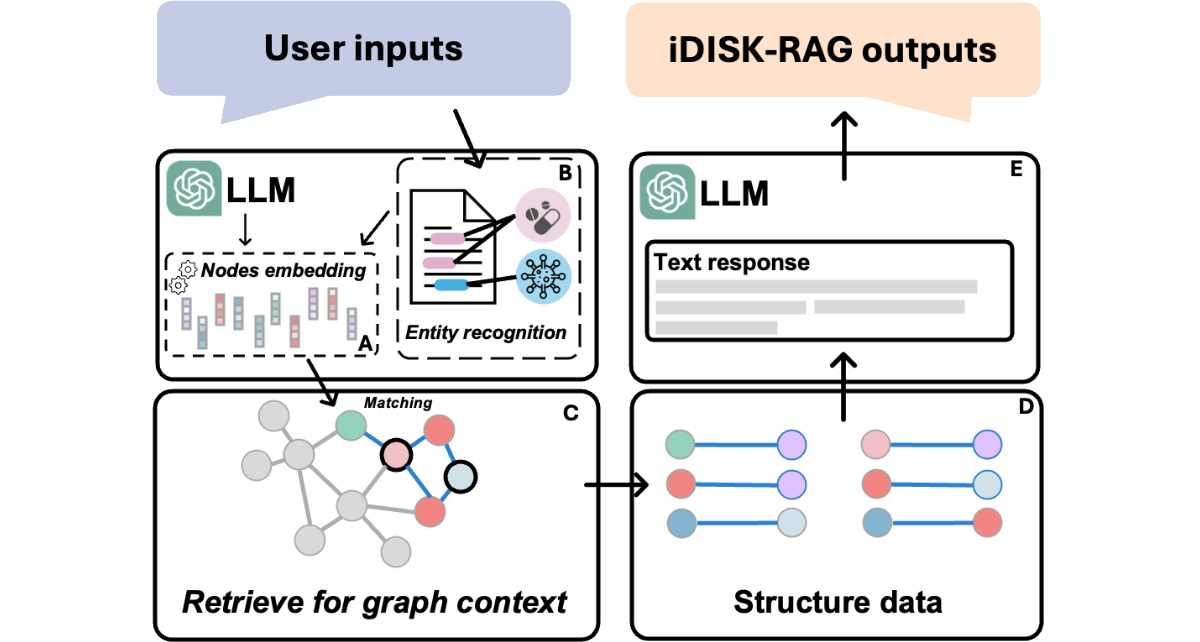
The overall design of the iDISK2.0-RAG (integrated Dietary Supplement Knowledgebase—retrieval-augmented generation). LLM: large language model.

Figure 2 illustrates the overall framework of iDISK2.0-RAG. We began by preprocessing all entities within the iDISK2.0 database, creating embedding vectors for each entity name using OpenAI’s embedding model, text-embedding-3-small (Figure 2A). This step allowed us to construct the iDISK2.0 entity vector database, which serves as a foundation for efficient and accurate entity matching. When a user inputs a query, we first extract relevant entities from the question by using an LLM (GPT-4.0; Figure 2B), using tailored prompts (see Multimedia Appendix 1 for specific prompts). The entities identified through this process are then transformed into corresponding vectors using the same OpenAI embeddings model. In the current implementation, relationships between entities are not embedded directly. Instead, we leverage the fixed and well-defined set of 6 relationship types in iDISK2.0 (eg, *is_effective_for* and *interacts_with*) to assign relationships based on the queried entity pairs. For instance, DSI-disease pairs are always associated with the *is_effective_for* relationship. This approach ensures computational efficiency while maintaining accuracy in retrieving relationship-based queries.

To match these identified entities with those in the iDISK2.0 database, we calculate the cosine similarity between the vectors of the identified entities and the vectors in the preprocessed iDISK2.0 entity vector database. Cosine similarity, a measure of similarity between two vectors in an inner product space, is calculated based on the cosine of the angle between them (detailed information on cosine similarity is provided in Multimedia Appendix 1). We then select the entity with the highest similarity score, provided that the score is 0.75 or higher, to ensure a reliable match between the identified entity and a corresponding entity in iDISK2.0.

Once a match is identified, the corresponding nodes are converted into Cypher queries, allowing for interaction with iDISK2.0, which is deployed on the Neo4j graph database platform. This process retrieves the relevant graph context (Figure 2C), which is returned as structured data in the form of triples (Figure 2D). Finally, an LLM is used to integrate the user’s original question with the retrieved structured data (triples) and generate a comprehensive and contextually enhanced response (Figure 2E; see Multimedia Appendix 1 for specific prompts).

### Evaluation

We extracted two types of questions from information on the MSKCC website: true-or-false questions and multiple-choice questions (MCQs). Specifically, we extracted information regarding the effectiveness of DSs and diseases and the interactions between DSs and drugs. This information was formatted into true-or-false questions and MCQs (473 true-or-false questions and 329 MCQs). To evaluate the performance of iDISK2.0-RAG and two LLMs (GPT-3.5 and GPT-4.0), we randomly selected 100 questions from each type (true-or-false questions and MCQs). These questions were answered by iDISK2.0-RAG, GPT-3.5, and GPT-4.0, and their accuracy was calculated based on the correct answers. This process was repeated 10 times using bootstrapping, resulting in each system answering 1000 true-or-false questions and 1000 MCQs. Accuracy metrics for each sampling iteration were then computed to derive the performance distribution. To ensure a fair evaluation, all knowledge from MSKCC was excluded from the retrieval stage in the RAG framework, preventing any influence of MSKCC-integrated knowledge on the test results. The rose plot was drawn using ChiPlot [27].

### RAG-Enhanced Intelligent Visual Interfaces

We developed a web-based graphical portal that allows users to access knowledge from iDISK2.0 intuitively and flexibly. Specifically, we built the backend (server-side) using Django (Django Software Foundation) [28], an advanced web framework based on Python (Python Software Foundation). The backend also hosts our iDISK2.0-RAG framework to provide users with more accurate retrieval information. The front end (web application side) is constructed using HTML5 and CSS, with JavaScript used for data interaction. Specifically, when the backend receives a query from the front end, the system first uses an LLM, such as GPT-4.0, to extract relevant entities from the user’s question through tailored prompts. These extracted entities are then matched with pretrained embedding information from a comprehensive entity vocabulary stored in the backend. Once a match is identified, the RAG system generates corresponding Cypher queries based on the matched entities. These Cypher queries are subsequently used to interact with iDISK2.0, which is deployed on the Neo4j graph database platform, to retrieve the relevant contextual subgraphs. The retrieved subgraphs provide structured data that are essential for generating an accurate response. In the final step, the LLM, guided by specific prompts, is used once again to transform the structured data retrieved by the RAG system into natural language, which is then returned to the front end’s dialogue interface. In cases where the entities mentioned in the user’s question cannot be successfully matched within iDISK2.0, the LLM (GPT-4.0) relies on its internal knowledge base to generate a corresponding answer, which is also delivered to the front end’s dialogue interface. This approach ensures that users receive comprehensive and contextually relevant responses, whether through structured data retrieval or the LLM’s generative capabilities.

### Ethical Considerations

This study exclusively used publicly available datasets from trusted sources, including NMCD, MSKCC, DSLD, and LNHPD, for the development and evaluation of iDISK2.0. The study did not involve human participants, and no personal, clinical, or proprietary data were accessed or used. Due to these reasons, this study did not require ethics board approval according to the University of British Columbia's Behavioral Research Ethics Board (BREB) guidelines [29]. The data collection and integration processes adhered to the terms and conditions of the respective data providers. For enhanced security, strict protocols were followed during data processing to prevent the inadvertent inclusion of sensitive information. The deployment and use of iDISK2.0 also comply with applicable ethical and legal standards to safeguard the privacy and integrity of the data.

## Results

### The Integrated Dietary Supplements Knowledge Base 2.0

Through systematic collection and coordination, we ultimately obtained 279 concepts of DSIs, 270 concepts of rugs, 231 concepts of diseases, and 425 concepts of symptoms from the latest version of the MSKCC website. From DSLD, we acquired 4318 concepts of DSIs and 92,651 concepts of DSPs. In addition, from the Natural Health Products database, we gathered 4690 concepts of DSIs and 71,364 concepts of DSPs. After conducting data management and data cleaning, we integrated data from the 3 sources through biomedical entity term normalization and knowledge integration. The iDISK 2.0 encompasses 174,317 entities across 7 types (Table 1), including 8091 DSI entities; 163,806 DSP entities; 786 disease entities; 625 drug entities; 425 symptom entities; 567 dietary therapeutic class (TC) entities; and 17 system organ class (SOC) entities. In addition, there are 6 types of relationships among the 7 entity types (Table 1), including DSP-DSI, DSI-disease, DSI-symptom, DSI-drug, DSI-TC, and DSI-SOC.

**Table 1:** The concepts, relationships, and attributes in iDISK2.0 (integrated Dietary Supplement Knowledgebase 2.0).

Data elements			iDISK^a^ (old version), n		iDISK (new version), n
**Concept**					
	Dietary supplement ingredient	4208		8091	
	Dietary supplement product	137,568		163,806	
	Drug	495		625	
	Disease	776		786	
	Therapeutic class	605		567	
	System organ class	17		17	
	Sign/symptoms	985		425	
	Total	144,654		174,317	
**Relationship**					
	is_effective_for	5363		5245	
	has_therapeutic_class	5454		4435	
	has_adverse_effect_on	3168		2598	
	has_adverse_reaction	2233		1342	
	has_ingredient	689,826		317,062	
	interacts_with	3631		3583	
	Total	709,675		334,265	
**Attribute**					
	Company name	—^b^		163,806	
	Company address	—		163,806	
	Product purpose	—		65,097	
	Product risk	—		63,623	
	Background	1399		1289	
	Safety	1219		1179	
	Mechanism of action	258		277	
	Source material	5532		4277	
	Interaction rating	3076		3086	
	Effectiveness rating	4307		4623	
	Total	15,791		471,063	

^a^iDISK: integrated Dietary Supplement Knowledgebase.

^b^Not applicable.

The iDISK 2.0 also includes 471,063 attributes (Table 1), comprising 163,806 DSP company names; 163,806 DSP company addresses; 65,097 DSP purposes; 63,623 DSP risks; 1289 DSI backgrounds; 1179 DSI safety profiles; 277 DSI mechanisms of action; 4277 DSI source materials; 3086 DSI-drug interaction ratings; and 4623 DSI-disease effectiveness ratings.

### An iDISK2.0-Based RAG System

To address the potential hallucination issue present in current LLMs and thereby improve the accuracy of user responses, we developed an iDISK2.0-based RAG system. This system integrates the strengths of KG and LLMs to efficiently retrieve relevant knowledge from iDISK and use the language model to generate feedback and facilitate user interaction.

**Figure 3:**
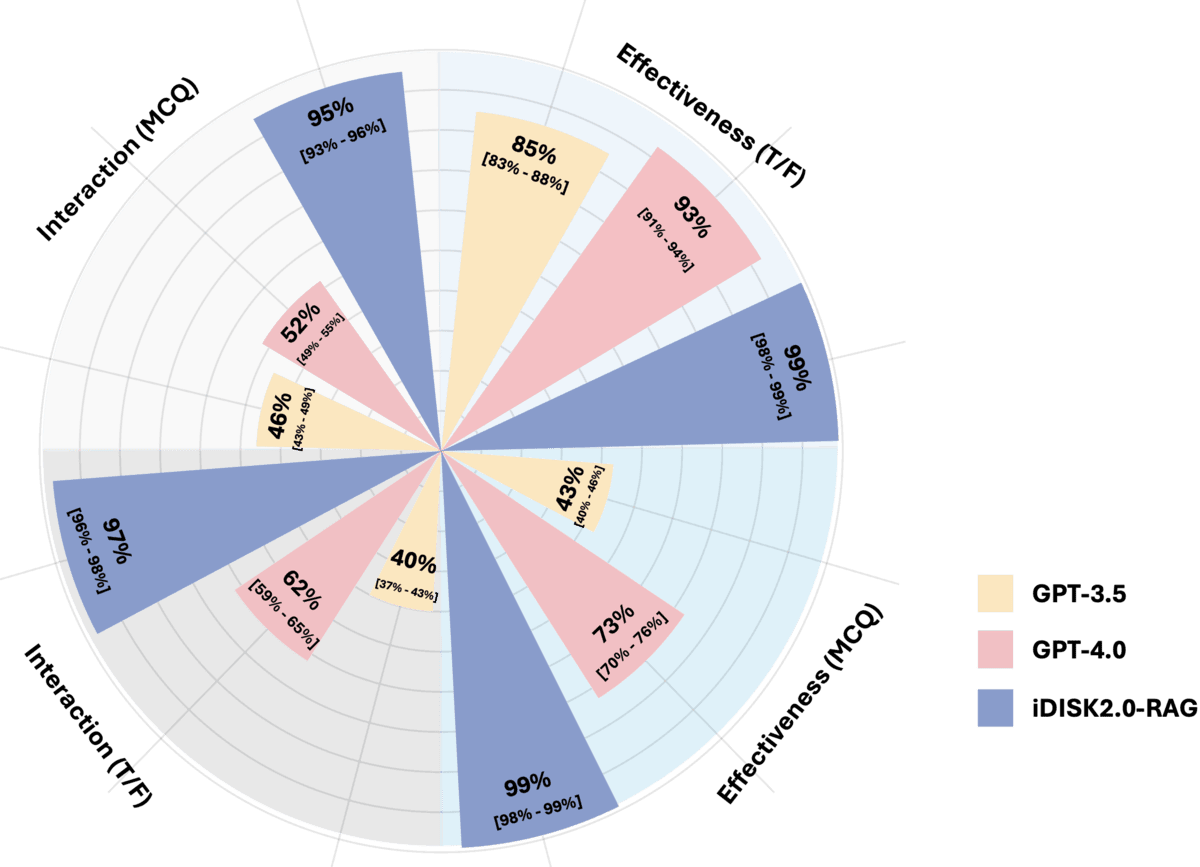
Model performance in question-and-answer tasks. T/F: true-or-false; MCQ: multiple-choice question; iDISK2.0-RAG: integrated Dietary Supplement Knowledgebase—retrieval-augmented generation.

We collected data from the MSKCC website regarding the effectiveness of dietary supplements and diseases and the interactions between DSs and drugs. From these data, we generated a total of 473 true-or-false questions and 329 MCQs. Specifically, there are 164 true-or-false questions, 123 MCQs related to DSs and diseases, and 309 true-or-false questions and 206 MCQs related to DSs and drugs. For instance, in the MSKCC database, there is a record stating, “Vitamin C is used to prevent and treat the common cold.” We used this record to generate corresponding true-or-false questions and MCQs for evaluative purposes. The true-or-false question derived from this statement is “Is it true that Vitamin C is effective for the common cold?” with the correct answer being “True.” In addition, we developed an MCQ to assess understanding: “Out of the given list, which disease is Vitamin C effective for?” The provided options are “Bladder stones, Common cold, Stroke, Bleeding hemorrhoids, and None of the above.” The correct answer is “Common cold.” Figure 3 illustrates the performance (accuracy) of two LLMs (GPT-3.5 and GPT-4.0) and the iDISK2.0-RAG system on true-or-false questions and MCQs related to DSs and diseases, as well as DSs and drugs. The results show that iDISK2.0-RAG achieves over 95% accuracy across all question types: 99% (990/1000) for true-or-false questions on DSs and diseases, 99% (993/100) for MCQs on DSs and diseases, 97% (974/1000) for true-or-false questions on DSs and drugs, and 95% (948/1000) for MCQs on DSs and drugs. In contrast, the performance of the two LLMs is notably lower, with GPT-4.0 outperforming GPT-3.5: GPT-4.0 achieved 93% (929/1000) accuracy on true-or-false questions related to DSs and diseases, compared with 85% (854/1000) for GPT-3.5; 73% (727/1000) accuracy on MCQs related to DSs and diseases, compared with 43% (426/1000) for GPT-3.5; 62% (618/1000) accuracy on true-or-false questions related to DSs and drugs, compared with 40% (400/1000) for GPT-3.5; and 52% (517/1000) accuracy on MCQs related to DSs and drugs, compared with 46% (457/1000) for GPT-3.5. Despite GPT-4.0’s superior performance over GPT-3.5, both models fall significantly short of the accuracy achieved by iDISK2.0-RAG in these specialized domains.

### A User-Friendly Intelligent User Interface for Knowledge Retrieval

**Figure 4:**
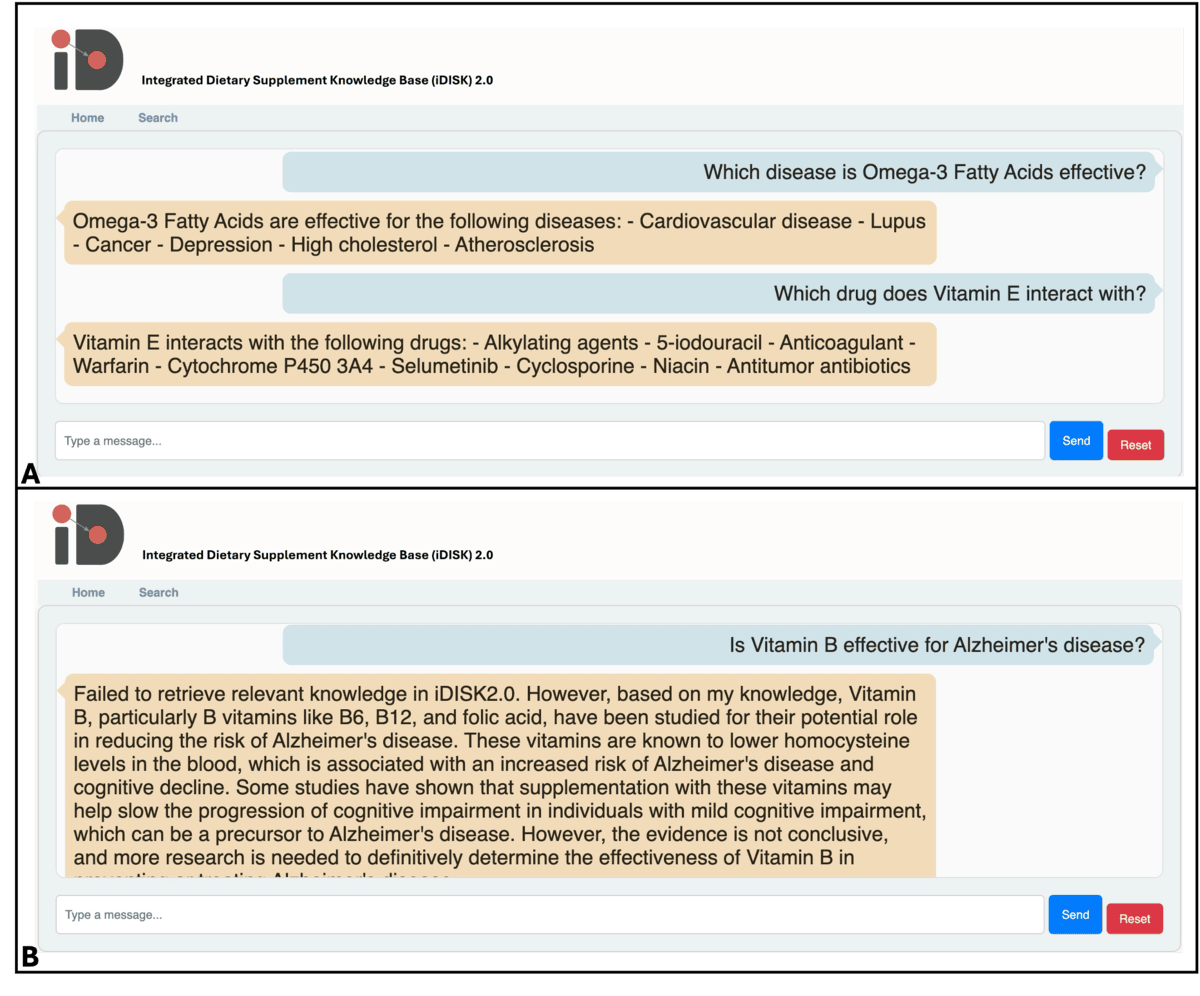
An example of question-and-answer on the user interface.

Knowledge retrieval is one of the most common application scenarios of BKG in biomedical research. In recent years, LLMs have shown outstanding performance in NLP tasks but suffer from the issue of “hallucinations,” especially within specific domains [15-17]. To facilitate the use of iDISK2.0 by biomedical and clinical researchers in DS studies, we developed a web portal based on iDISK2.0-RAG as the backend. This RAG-based user interface allows users to input free-form text queries, generating meaningful biomedical text responses based on the established knowledge within iDISK2.0 to answer users’ questions. Figure 4A illustrates an example of a user query using free-form text. When the user inputs a question such as “Which disease is Omega-3 Fatty Acids effective for?” the user interface retrieves relevant knowledge from the iDISK2.0-RAG backend and returns the corresponding answer: “Omega-3 Fatty Acids are effective for: - Cardiovascular disease - Lupus - Cancer - Depression - High cholesterol - Atherosclerosis.” In cases where the user’s query cannot be answered directly from the iDISK2.0 knowledge base, the RAG framework informs the user that the specific knowledge is not available within iDISK2.0. Subsequently, the LLM within the framework attempts to answer the question based on its own knowledge, as depicted in Figure 4B.

## Discussion

### Principal Findings

iDISK integrates information from 4 well-designed DS databases, ensuring it contains the most comprehensive DS-related information available. iDISK2.0 inherits this feature, maintaining its status as the most thorough DS information repository. As source resources are updated, much DS-related information has been upgraded. For instance, when the DSLD was first launched in 2013, it contained nearly 17,000 label entries; now, it includes nearly 190,000, an increase of more than tenfold in just a decade. In addition, many DS details have been corrected; for example, the older version of iDISK included an ingredient from DSLD labeled “Araceae,” which was inaccurate. In the latest version of DSLD, this ingredient has been removed. The observed reduction in the number of entities, such as in the “signs/symptoms” category, further reflects the enhanced data integration and noise reduction strategies used in iDISK2.0. By consolidating duplicate entries, refining entity definitions, and filtering out ambiguous or irrelevant information, iDISK2.0 significantly improves the overall quality of the knowledge base. Upgrading iDISK to iDISK2.0 is crucial to maintaining the accuracy and relevance of DS information. Without regular updates, the knowledge base could become outdated, leading to potential misinformation and inefficiencies in DS-related research and health care decisions. The continuous integration of the latest data and refined processes in iDISK2.0 helps mitigate these risks, ensuring that users have reliable, current information to make informed decisions about dietary supplements.

LLMs like GPT-4.0 have significantly advanced the field of natural language processing. However, they still exhibit notable limitations, particularly in domain-specific applications such as DS information retrieval. A key challenge is their propensity for generating “hallucinations”—plausible yet incorrect information—stemming from their reliance on extensive but often unverified data sources [18]. This issue is particularly problematic when precision is critical. For example, our evaluation highlighted a case involving the use of coenzyme Q10, also known as ubiquinone, which is documented for its effectiveness in preventing migraines according to the MSKCC database. To test our system’s accuracy, we posed an MCQ: “Which disease is Ubiquinone effective for?” with options including migraines, hypertension, menstrual disorders, cholestasis, and none of the above, despite the correct answer being “Migraines,” based on robust evidence. However, GPT-4.0 returned “Cholestasis” as the answer, even though only limited studies have explored the relationship between coenzyme Q10 and intrahepatic cholestasis of pregnancy [30,31], with no definitive conclusions indicating its efficacy in this context. In contrast, numerous studies have established the effectiveness of coenzyme Q10 in the prevention and treatment of migraines [32-34]. This error underscores a critical concern: while LLMs have vast knowledge bases, they can still falter by either misinterpreting context or lacking explicit, well-established information.

To address these challenges, we developed iDISK2.0 and integrated it with an RAG system. This integration combines the expansive knowledge base of LLMs with the precise, validated data from iDISK2.0, significantly reducing the risk of hallucinations. In testing, the RAG system, drawing from verified knowledge within iDISK2.0, consistently provided accurate answers, such as correctly identifying coenzyme Q10’s efficacy for migraines. While the current evaluation focused on true-or-false questions and MCQs derived from the MSKCC database to ensure controlled and reproducible testing, we recognize that real-world, open-ended queries often require more nuanced and context-sensitive responses. Our evaluation results indicate that iDISK2.0-RAG achieves over 95% accuracy when retrieving structured knowledge, whereas stand-alone LLMs perform substantially worse, with GPT-4.0 achieving only 62% (618/1000) accuracy in true-or-false questions and 52% (517/1000) in MCQs on DS-drug interactions. This underscores the importance of structured knowledge retrieval in mitigating misinformation. However, instances where the system relies solely on the LLM’s generative capabilities without sufficient retrieval support may still be prone to inaccuracies. To address the limitations of structured retrieval and enhance real-world applicability, we plan to develop a more comprehensive BKG that integrates diverse data sources beyond iDISK2. This expanded BKG will include biomedical literature to summarize the evidence [35], social media data to capture public perceptions, electronic health records to extract evidence of efficacy and safety [36-38], and postmarket surveillance data to address safety concerns [39]. By leveraging this holistic BKG, we aim to enhance the system’s capability to interpret and respond to real-world, open-ended queries. Future evaluations will incorporate open-ended questions collected from diverse user groups and assess both quantitative metrics, such as accuracy, and qualitative metrics, such as user satisfaction. This integrated approach will provide deeper insights into the system’s robustness and applicability. Further enhancing this solution, we incorporated a user-friendly interface with the iDISK2.0-RAG backend, allowing users to access accurate DS information through simple conversational text inputs. This setup not only facilitates better-informed decisions but also improves the overall user experience by ensuring the dissemination of the most accurate and current DS information. The necessity of this innovation is emphasized by the potential risks of relying solely on LLMs, which can propagate misinformation and lead to inefficiencies in both research and health care decisions. Moving forward, future research should focus on refining the interaction between LLMs and RAG systems. A potential avenue could involve mechanisms that allow LLMs to defer to the more reliable RAG output in cases where the evidence is more compelling. This approach would significantly enhance the accuracy and trustworthiness of artificial intelligence–generated responses in biomedical applications.

Despite adopting stricter mapping and integration schemes compared with the older version of iDISK, some errors and noise still exist in the final results. These inaccuracies partly stem from the imprecise expressions in the source databases. In addition, errors arise when attempting to map entities from the source databases to UMLS CUIs for subsequent entity integration. The entity extraction and mapping software QuickUMLS sometimes returns incorrect results. For instance, the MSKCC database records an interaction between “Andrographis” and “Blood pressure-lowering drugs”: “Andrographis interacts with Blood pressure-lowering drugs.” When extracting entities from this knowledge, we extracted the drug entity “Blood pressure-lowering drugs” from the text. However, QuickUMLS mapped “Blood pressure-lowering drugs” to “Drug (UMLS CUI: C0013227),” resulting in incorrect mapping and introduction of errors into the integration process.

Given the current limitations of this study, we plan to implement different strategies in the future to further reduce errors. For instance, we can introduce entity extraction techniques based on LLMs to extract DS-related information stored in text. For example, we may employ various prompt strategies, including rule-based prompts (eg, HealthPrompt [40]), hard-prompt learning [41], and soft-prompt learning [42]. In addition, we can leverage LLMs to batch-check entity extraction and mapping results and perform quality control on the integration outcomes. Recent research has highlighted the beneficial applications of BKGs in the medical field [7,43]. Furthermore, the rise of LLMs has spurred discussions on how best to combine the strengths of these models and BKGs [44]. In the future, besides continuously maintaining iDISK to ensure it remains the most up-to-date and comprehensive DS information resource, we will also explore ways to better assist users in using iDISK. For example, we plan to introduce knowledge graph embedding technology. By using graph-structured reasoning, we aim to offer reasonable predictions for knowledge not yet included in existing databases, thereby helping users discover more information. This approach will not only enhance the precision of user queries but also expand the scope of knowledge available to them. To ensure the long-term reliability and accuracy of iDISK2.0, we plan to establish a pipeline for annual updates integrating new data from trusted sources, such as NMCD, MSKCC, DSLD, and LNHPD. This includes automated data extraction, preprocessing, and stringent quality control measures. In addition, we will continuously adapt the system’s embedding strategy and retrieval mechanisms to align with the evolution of commercial LLMs. The open-source nature of iDISK2.0 will enable contributions from the research community, fostering collaborative improvements. Regular evaluations with updated test sets will further ensure the sustained performance of the RAG system.

The modular design of the iDISK2.0-RAG framework facilitates its adaptability to other areas of health care beyond dietary supplements. BKGs have shown significant promise in facilitating complex biomedical processes such as drug discovery [45]. By integrating domain-specific knowledge bases, such as those focused on drug interactions or general medical advice, the framework could be extended to address broader biomedical applications. For example, incorporating drug interaction databases or broader biomedical ontologies could enable the system to handle queries related to drug-drug interactions, disease management, and care pathways. Future work will explore these possibilities, evaluating the system’s performance across diverse domains and refining its capabilities to support a wider range of health care–related tasks.

### Conclusion

In this study, we developed iDISK2.0, an updated repository for DS information, integrating data from NMCD, MSKCC, DSLD, and LNHPD. To address the limitations of LLMs, we introduced an RAG system combining LLMs with a BKG for accurate DS queries. iDISK2.0-RAG outperformed stand-alone LLMs, achieving over 95% accuracy. While some challenges remain with source data and entity mapping, future efforts will focus on improving entity extraction and quality control, ensuring iDISK2.0 continues to support informed DS decision-making. In addition, the adaptable design of the iDISK2.0-RAG framework offers significant potential for expansion into broader health care domains, such as drug interactions and general medical advice.

## Appendix

Multimedia Appendix 1 Unified Medical Language System (UMLS) semantic type restrictions, framework prompts, and cosine similarity method for entity matching.

## Abbreviations

API: application programming interface
BKG: biomedical knowledge graph
CUI: Concept Unique Identifier
DS: dietary supplement
DSI: dietary supplement ingredient
DSLD: Dietary Supplement Label Database
DSP: dietary supplement product
iDISK: integrated Dietary Supplement Knowledgebase
iDISK2.0: integrated Dietary Supplement Knowledgebase 2.0
iDISK2.0-RAG: integrated Dietary Supplement Knowledgebase—retrieval-augmented generation
KG: knowledge graph
LLM: large language model
LNHPD: Licensed Natural Health Products Database
MCQ: multiple-choice question
MSKCC: Memorial Sloan Kettering Cancer Center
NMCD: Natural Medicines Comprehensive Database
RAG: retrieval-augmented generation
SOC: system organ class
TC: therapeutic class
UMLS: Unified Medical Language System

## Data Availability

The datasets generated during this study are available in the iDISK2.0 (integrated Dietary Supplement Knowledgebase 2.0) repository on GitHub [46].

## References

[1] (1994). Dietary Supplement Health and Education Act of 1994. National Institutes of Health.

[2] (2024). Dietary supplements. US Food and Drug Administration.

[3] (2023). Nine in ten dietary or nutritional supplement users agree that dietary supplements are essential to maintaining their health. Ipsos.

[4] (2024). CRN responds to recent JAMA commentary on multivitamin efficacy. CRN.

[5] Dwyer JT, Coates PM (2018). Why Americans need information on dietary supplements. J Nutr.

[6] Geller AI, Shehab N, Weidle NJ, Lovegrove MC, Wolpert BJ, Timbo BB, Mozersky RP, Budnitz DS (2015). Emergency department visits for adverse events related to dietary supplements. N Engl J Med.

[7] Su C, Hou Y, Zhou M, Rajendran S, Maasch JRA, Abedi Z, Zhang H, Bai Z, Cuturrufo A, Guo W, Chaudhry FF, Ghahramani G, Tang J, Cheng F, Li Y, Zhang R, DeKosky ST, Bian J, Wang F (2023). Biomedical discovery through the integrative biomedical knowledge hub (iBKH). iScience.

[8] Rubin DL, Shah NH, Noy NF (2008). Biomedical ontologies: a functional perspective. Brief Bioinform.

[9] Zhu Y, Elemento O, Pathak J, Wang F (2019). Drug knowledge bases and their applications in biomedical informatics research. Brief Bioinform.

[10] Rizvi R, Vasilakes J, Adam TJ, Melton GB, Bishop JR, Bian J, Tao C, Zhang R (2020). iDISK: the integrated DIetary supplements knowledge base. J Am Med Inform Assoc.

[11] (2019). Natural Medicines Comprehensive Database (NMCD). NatMed Pro.

[12] (2021). About herbs, botanicals & other products. Memorial Sloan Kettering Cancer Center.

[13] (2020). Dietary Supplement Label Database (DSLD). National Institutes of Health.

[14] (2021). Licensed Natural Health Products Database (LNHPD).

[15] Yao JY, ing KP, Liu ZH, Ning MN, Liu YY, Yuan L (2023). LLM Lies: hallucinations are not bugs, but features as adversarial examples. ArXiv. Preprint posted online on October 2, 2023.

[16] Xu Z (2024). Hallucination is inevitable: an innate limitation of large language models. ArXiv. Preprint posted online on January 22, 2024.

[17] Rawte V, Sheth A, Das A (2023). A survey of hallucination in large foundation models. ArXiv. Preprint posted online on September 12, 2023.

[18] Hou Y, Yeung J, Xu H, Su C, Wang F, Zhang R (2023). From answers to insights: unveiling the strengths and limitations of ChatGPT and biomedical knowledge graphs. medRxiv. Preprint posted online on June 12, 2023.

[19] Zakka C, Shad R, Chaurasia A, Dalal AR, Kim JL, Moor M, Fong R, Phillips C, Alexander K, Ashley E, Boyd J, Boyd K, Hirsch K, Langlotz C, Lee R, Melia J, Nelson J, Sallam K, Tullis S, Vogelsong MA, Cunningham JP, Hiesinger W (2024). Almanac - retrieval-augmented language models for clinical medicine. NEJM AI.

[20] Li H, Su Y, Cai D, Wang Y, Liu L (2022). A survey on retrieval-augmented text generation. ArXiv. Preprint posted online on February 2, 2022.

[21] Gao Y, Xiong Y, Gao X, Jia K, Pan J, Bi Y, Dai Y, Sun J (2024). Retrieval-augmented generation for large language models: a survey. SSRN.

[22] Sanmartin D (2024). KG-RAG: bridging the gap between knowledge and creativity. ArXiv. Preprint posted online on May 20, 2024.

[23] Xu Z, Jerome Cruz M, Guevara M, Wang T, Deshpande M, Wang X, Li Z (2024). Retrieval-augmented generation with knowledge graphs for customer service question answering.

[24] Soldaini L, Goharian N (2016). Quickumls: a fast, unsupervised approach for medical concept extraction. MedIR Workshop.

[25] Bodenreider O (2004). The unified medical language system (UMLS): integrating biomedical terminology. Nucleic Acids Res.

[26] (2013). Neo4j graph database and analytics.

[27] ChiPlot.

[28] (2013). Django.

[29] UBC Clinical Research Ethics General Guidance Notes. The University of British Columbia.

[30] Martinefski MR, Rodriguez MR, Buontempo F, Lucangioli SE, Bianciotti LG, Tripodi VP (2020). Coenzyme Q 10 supplementation: a potential therapeutic option for the treatment of intrahepatic cholestasis of pregnancy. Eur J Pharmacol.

[31] Martinefski MR, Cocucci SE, Di Carlo MB, Vega HR, Lucangioli SE, Perazzi BE, Tripodi VP (2020). Fetal coenzyme Q10 deficiency in intrahepatic cholestasis of pregnancy. Clin Res Hepatol Gastroenterol.

[32] Yaghini O, Hoseini N, Ghazavi MR, Mansouri V, Nasiri J, Moosavian T, Salehi MM (2022). A comparative study on the efficacy of coenzyme Q10 and amitriptyline in the prophylactic treatment of migraine headaches in children: a randomized controlled trial. Adv Biomed Res.

[33] Sándor PS, Di Clemente L, Coppola G, Saenger U, Fumal A, Magis D, Seidel L, Agosti RM, Schoenen J (2005). Efficacy of coenzyme Q10 in migraine prophylaxis: a randomized controlled trial. Neurology.

[34] Shoeibi A, Olfati N, Soltani Sabi M, Salehi M, Mali S, Akbari Oryani M (2017). Effectiveness of coenzyme Q10 in prophylactic treatment of migraine headache: an open-label, add-on, controlled trial. Acta Neurol Belg.

[35] Schutte D, Vasilakes J, Bompelli A, Zhou Y, Fiszman M, Xu H, Kilicoglu H, Bishop JR, Adam T, Zhang R (2022). Discovering novel drug-supplement interactions using SuppKG generated from the biomedical literature. J Biomed Inform.

[36] Zhan Z, Zhou S, Li M, Zhang R (2025). RAMIE: retrieval-augmented multi-task information extraction with large language models on dietary supplements. J Am Med Inform Assoc.

[37] Fan Y, Zhang R (2018). Using natural language processing methods to classify use status of dietary supplements in clinical notes. BMC Med Inform Decis Mak.

[38] Fan Y, Zhou S, Li Y, Zhang R (2021). Deep learning approaches for extracting adverse events and indications of dietary supplements from clinical text. J Am Med Inform Assoc.

[39] Vasilakes J, Rizvi RF, Zhang J, Adam TJ, Zhang R (2019). Detecting signals of dietary supplement adverse events from the CFSAN adverse event reporting system (CAERS). AMIA Jt Summits Transl Sci Proc.

[40] Sivarajkumar S, Wang Y (2022). HealthPrompt: a zero-shot learning paradigm for clinical natural language processing. AMIA Annu Symp Proc.

[41] Shin T, Razeghi Y, Logan IV RL, Wallace E, Singh S (2020). AutoPrompt: eliciting knowledge from language models with automatically generated prompts.

[42] Lester B, Al-Rfou R, Constant N (2021). The power of scale for parameter-efficient prompt tuning.

[43] Xiao Y, Hou Y, Zhou H, Diallo G, Fiszman M, Wolfson J, Zhou L, Kilicoglu H, Chen Y, Su C, Xu H, Mantyh WG, Zhang R (2024). Repurposing non-pharmacological interventions for alzheimer's disease through link prediction on biomedical literature. Sci Rep.

[44] Yan Y, Hou Y, Xiao Y, Zhang R, Wang Q (2024). KNowNEt: Guided health information seeking from LLMs via knowledge graph integration. IEEE Trans Vis Comput Graph.

[45] Su C, Hou Y, Wang F (2022). NN-based biomedical knowledge graph mining in drug development. Graph Neural Networks: Foundations, Frontiers, and Applications.

[46] iDISK 2.0 - integrated Dietary Supplement Knowledgebase 2.0. GitHub.

