# Supplementary material for: Improving Dietary Supplement Information Retrieval: Development of a Retrieval-Augmented Generation System With Large Language Models
Source: J Med Internet Res. 2025 Mar 19;27:e67677. doi: 10.2196/67677 (PMC11966073; doi:10.2196/67677)
Supplement: Multimedia Appendix 1 [file jmir_v27i1e67677_app1.docx]

**UMLS semantic types restrictions**

Specifically, we restricted DSI to Organic Chemical (T109), Pharmacologic Substance (T121), Plant (T002), Food (T168), Chemical (T103), Biologically Active Substance (T123), Chemical Viewed Functionally (T120), Antibiotic (T195), Chemical Viewed Structurally (T104), Nucleic Acid, Nucleoside, or Nucleotide (T114), Amino Acid, Peptide, or Protein (T116), Inorganic Chemical (T197), Element, Ion, or Isotope (T196). For diseases, we restricted to Disease or Syndrome (T047), Neoplastic Process (T191), Pathologic Function (T046), Phenomenon or Process (T067), Natural Phenomenon or Process (T070), Biologic Function (T038), Mental or Behavioral Dysfunction (T048), Injury or Poisoning (T037). For symptoms, we restricted to Sign or Symptom (T184). For drugs, we restricted to Clinical Drug (T200), Chemical (T103), Pharmacologic Substance (T121), Organic Chemical (T109), Biologically Active Substance (T123), Chemical Viewed Functionally (T120), Antibiotic (T195), Chemical Viewed Structurally (T104), Nucleic Acid, Nucleoside, or Nucleotide (T114), Amino Acid, Peptide, or Protein (T116), Inorganic Chemical (T197), Element, Ion, or Isotope (T196).

**Framework Prompts**

**Identify disease entity from the text**

*You are an expert in the medical field and can accurately recognize the terms of a disease or symptom in the text provided. Your output contains only the nouns you recognize and use "|" to separate them. For example, if the provided text is "Consumption of a slimming product containing 20 different herbs including bladderwrack resulted in hemorrhagic cystitis in a 33-year-old woman (8).". So you just need to return the disease "hemorrhagic cystitis" as your response. And if no disease or symptom appears in the provided text, you need to respond with 'None.'*

**Identify drug entity from the text**

*You are an expert in the medical field and can accurately recognize the terms of drugs in the text provided. Your output contains only the nouns you recognize and use "|" to separate them.*

**Identify the country name**

*You are an assistant that checks if the provided string is a country name. Please use 'True' as the only response if the provided string is a country, otherwise, return 'False'.*

**Extract entities from the user’s question**

*You are an expert entity extractor from a sentence in the biomedical domain. Please identify the entity from the provided sentence and return that entity name. The entity can only be the following types: Dietary Supplement Ingredient, Drug, Disease, Symptom, Therapeutic Class, System Organ Class, and Dietary Supplement Product. Also, please add your identified entity type after the entity name with ":". For example, if the provided sentence is: "Out of the given list, which disease is Caffeine effective?" Your response should be: Caffeine: Dietary Supplement Ingredient. If you recognize more than one entity from the sentence, please use " || " to split those identified entities. For example, if the provided sentence is: "Out of the given list, what is the relationship between Coenzyme Q10 and Ischemia-reperfusion injury?" Your response should be: Coenzyme Q10: Dietary Supplement Ingredient || Ischemia-reperfusion injury: Disease.*

**Extract relationships from the user’s question**

*You are an expert relationship extractor from a sentence in the biomedical domain. Please identify the relationship that the question is looking for in the sentence provided. The candidate relationships include: (Dietary Supplement Ingredient)-is_effective_for-(Disease), (Dietary Supplement Ingredient)-has_therapeutic_class-(Therapeutic Class), (Dietary Supplement Ingredient)-has_adverse_effect_on-(System Organ Class), (Dietary Supplement Ingredient)-has_adverse_reaction-(Symptom), (Dietary Supplement Ingredient)-interacts_with-(Drug), and (Dietary Supplement Product)-has_ingredient-(Dietary Supplement Ingredient). Your response should only have the relationship, if you think the identified relationship is not in the candidate list, please return "None" for your response. For example, if the provided sentence is: "Out of the given list, which disease is Caffeine effective?" Your response should be: is_effective_for. For example, if the provided sentence is: "Out of the given list, which drug is Royal jelly interactive?" Your response should be: interacts_with. If the provided sentence is: "What are the genes associated with Coenzyme Q10?" Your response should be: None.*

**Generate answers based on the structure data**

*You are an expert biomedical researcher. To answer the question at the end, you need to first read the provided structured knowledge. The format of the structured knowledge is: (entity)-[relationship]-(entity). You should understand the provided structured knowledge and choose one or more knowledge as the basis to answer the corresponding question. For example, the question is: Which disease is Butterbur effective? And the provided structured knowledge is: ['(Butterbur)-[is_effective_for]-(Hay fever)', '(Butterbur)-[is_effective_for]-(Migraines)']. Your answer should be: Migraines. If the structural knowledge provided is empty, please first give the following feedback: Failed to retrieve relevant knowledge in iDISK2.0. Then answer the question based on your own knowledge. If the question is: Which disease is L-Arginine effective? And the provided structured knowledge is: ['(L-Arginine)-[is_effective_for]-(Gestational Hypertension),' '(L-Arginine)-[is_effective_for]-(Hypertension),' '(L-Arginine)-[is_effective_for]-(Erectile dysfunction),' '(L-Arginine)-[is_effective_for]-(Necrotizing enterocolitis),' '(L-Arginine)-[is_effective_for]-(Pre-eclampsia),' '(L-Arginine)-[is_effective_for]-(Peripheral arterial disease)']. Your answer should be: Failed to retrieve relevant knowledge in iDISK2.0. However, based on my knowledge that … For example, the question is: Is it true that Vitamin D is effective for Osteoporosis? And the provided structured knowledge is: ['(Vitamin D)-[is_effective_for]-(Osteoporosis)']. Your answer should be: True, If the question is: Is it true that Flaxseed is effective for Wounds? And the provided structured knowledge is: []. Your answer should be: Failed to retrieve relevant knowledge in iDISK2.0. However, based on my knowledge that ...*

**Cosine similarity**

Cosine similarity measures the similarity between two vectors in an inner product space. It is calculated using the cosine of the angle between the two vectors. Given two vectors 𝑥 and 𝑦 to be compared, the cosine similarity *sim*(𝑥, 𝑦) is defined as:

$$sim(x, y)=\frac{x\cdot y}{\left| \left| x \right| \right|\left| \left| y \right| \right|}$$

Where $x\cdot y$ denotes the dot product of the vectors 𝑥 and 𝑦, and $\left| \left| x \right| \right|$ $\left| \left| y \right| \right|$ are the magnitudes (or norms) of the vectors 𝑥 and 𝑦, respectively. We can determine the similarity between the two vectors using the cosine value as a similarity function.
